# Prediction of High-Grade Vesicoureteral Reflux after Pediatric Urinary Tract Infection: External Validation Study of Procalcitonin-Based Decision Rule

**DOI:** 10.1371/journal.pone.0029556

**Published:** 2011-12-28

**Authors:** Sandrine Leroy, François Bouissou, Anna Fernandez-Lopez, Metin K. Gurgoze, Kyriaki Karavanaki, Tim Ulinski, Silvia Bressan, Geogios Vaos, Pierre Leblond, Yvon Coulais, Carlos Luaces Cubells, A. Denizmen Aygun, Constantinos J. Stefanidis, Albert Bensman, Liviana DaDalt, Stefanos Gardikis, Sandra Bigot, Dominique Gendrel, Gérard Bréart, Martin Chalumeau

**Affiliations:** 1 Centre for Statistics in Medicine, Oxford, United Kingdom; 2 Epidemiology of Emerging Diseases Unit, Institut Pasteur, Paris, France; 3 Inserm U953 Unit, Paris, France; 4 Department of Pediatrics, Paris Descartes University, Necker Hospital, AP-HP, Paris, France; 5 Department of Pediatrics, Children's Hospital, Paul Sabathier University, CHU Purpan, Toulouse, France; 6 Department of Pediatrics, Hospital Universitari Arnau de Vilanova, Lleida. Spain; 7 Firat University Faculty of Medicine, Elazig, Turkey; 8 Department of Nephrology "A. and P. Kyriakou" Childen's Hospital, Athens, Greece; 9 Department of Pediatric Nephrology, Trousseau Hospital and University Pierre et Marie Curie, Paris, France; 10 Department of Pediatrics, University of Padova, Padova, Italy; 11 Department of Pediatric Surgery, Alexandroupolis University Hospital, Thrace, Greece; 12 Department of Pediatrics, Jeanne de Flandre Hospital, Lille, France; 13 Department of Pediatrics, Hospital San Joan de Déu., Barcelona, Spain; John Hopkins Bloomberg School of Public Health, United States of America

## Abstract

**Background:**

Predicting vesico-ureteral reflux (VUR) ≥3 at the time of the first urinary tract infection (UTI) would make it possible to restrict cystography to high-risk children. We previously derived the following clinical decision rule for that purpose: cystography should be performed in cases with ureteral dilation and a serum procalcitonin level ≥0.17 ng/mL, or without ureteral dilatation when the serum procalcitonin level ≥0.63 ng/mL. The rule yielded a 86% sensitivity with a 46% specificity. We aimed to test its reproducibility.

**Study Design:**

A secondary analysis of prospective series of children with a first UTI. The rule was applied, and predictive ability was calculated.

**Results:**

The study included 413 patients (157 boys, VUR ≥3 in 11%) from eight centers in five countries. The rule offered a 46% specificity (95% CI, 41–52), not different from the one in the derivation study. However, the sensitivity significantly decreased to 64% (95%CI, 50–76), leading to a difference of 20% (95%CI, 17–36). In all, 16 (34%) patients among the 47 with VUR ≥3 were misdiagnosed by the rule. This lack of reproducibility might result primarily from a difference between derivation and validation populations regarding inflammatory parameters (CRP, PCT); the validation set samples may have been collected earlier than for the derivation one.

**Conclusions:**

The rule built to predict VUR ≥3 had a stable specificity (ie. 46%), but a decreased sensitivity (ie. 64%) because of the time variability of PCT measurement. Some refinement may be warranted.

## Introduction

During the past decade, many European and American pediatric societies have recommended that all young children undergo a cystography after a first febrile UTI [1], [2]. This systematic strategy is driven by the belief that VUR, especially moderate and high-grade VUR, is a risk factor for recurrent UTI, then renal scarring and long-term complications, such as recurrent infection, hypertension, poor renal growth, and eclampsia [3]. The systematic strategy is also based on the premise that VUR can be treated and/or children followed to keep them free of complications [1], [2]. However, even if systematic cystography offered a nearly 100% sensitivity to identify reflux, it is a truly non-selective strategy, meaning that many children undergo an unnecessary cystography, which is painful, irradiating and expensive, and increases the risk of iatrogenic UTI [4]. Regarding recent publications minimizing the clinical consequences of low-grade VUR [5], [6], the low rate of high-grade VUR (only 10% of the children with a first febrile UTI [7]), and the current discussions on the efficacy of high-grade VUR treatment [6], [8], [9], some new guidelines have proposed to never perform a cystography after a first febrile UTI in children 2 to 24 months old [10], [11]. However, these new guidelines have raised some concerns because of the risk of delaying high-grade VUR diagnosis [10]. Moreover, definite evidence about the absence of benefit of antibiotic prophylaxis is still lacking for many clinical situations because of methodological weaknesses of the available trials, and this could make the pendulum swing back towards specific indications of VUR treatment that have to be diagnosed accurately [12]. Furthermore, an American study of clinical practices evaluation measured that only 40% of children who would have benefited from a cystography actually underwent this examination after a first UTI, indicating that a more situational approach would likely be welcomed by parents and clinicians [13].

We believed, then, that there is scope for an evidence-based strategy, one that offered a moderate alternative to two diametrically opposed policies (cystography for all or no children). We derived a predictive tool for moderate and high-grade VUR (grade ≥3), aiming to avoid *a posteriori* unnecessary cystographies and not to miss those patients with high-grade VUR [14]. This predictive decision rule includes procalcitonin (PCT), a validated and sensitive predictor of VUR [15]–[17], and the ureteral dilation from renal ultrasonography (US) [18]. PCT was initially studied as a predictor of VUR because this sensitive biomarker was demonstrated to correlate with acute pyelonephritis and scars, both related to VUR [19], [20]. The rule indicates that in children between one month and four years old with a first febrile UTI, a cystography should be performed in cases with ureteral dilation (i.e. ureter visible on the renal ultrasonography – US) and a serum PCT level (measured at the time of UTI diagnosis) ≥0.17 ng/mL, or without ureteral dilatation (i.e. ureter not visible on the renal US) when the serum PCT level ≥0.63 ng/mL [14]. This rule yielded 86% (95%CI: 74–93) sensitivity with 47% (95%CI: 42–51) specificity; the internal mathematical validation confirmed the derivation predictive ability. However, four steps are involved in the development of a clinical decision rule: the creation of the rule, internal validation, external reproducibility, and assessment of its impact on clinical behavior [21]. Our aim was to evaluate the reproducibility of the prediction rule that we have developed and to perform its external validation.

## Results

### Patients' characteristics

Eight centres were included [18], [22]–[31]. In all centres, patients underwent a radiological cystography, and had a PCT measurement with the LUMItest PCT immunoluminometric assay or the BRAHMS PCT-Q semiquantitative rapid test (BRAHMS AG, Hennigsdorf, Germany). Half of the centres collected urine with suprapubic aspiration or urethral catheterization for non-toilet trained children, and half used sterile bags (Table 1).

**Table 1:** Population characteristics according to each center.

Centre*	Urine collection techniques (threshold of the positive bacteriuria)†	n	Male n (%)	Age median (IQR)	All-grade VUR n (%)	Grade ≥3 VUR n (%)	CRP Median (IQR)
***Centres using SA or UC (n = 199)***							
Alex.	SA (any), UC (10^4^), CVM (10^5^)	40	9 (23)	10.5 (6.5–12.5)	12 (30)	8 (20)	57.0 (14.5–91.0)
Athens	SA (10^3^), UC (10^4^), CVM (10^5^)	52	26 (50)	6.6 (3.0–9.8)	10 (19)	0 (0)	42.4 (6.1–108)
Barcelona	SA (any), UC (5.10^4^), CVM (10^5^)	55	22 (41)	6.0 (3.0–9.0)	13 (24)	4 (7)	44.6 (14.1–76.7)
Elazig	UC (10^3^), CVM (10^5^)	52	25 (48)	6.0 (6.0–36.0)	3 (6)	0 (0)	12.5 (4–40)
***Centres using SB (n = 213)***							
Lille	SB (10^5^)	23	7 (29)	8.5 (4.0–19.0)	14 (58)	3 (13)	52.5 (19–83)
Padova	SB (10^5^)	47	17 (38)	6.4 (3.1–11.4)	9 (19)	2 (4)	61.0 (37–120)
Paris	SB (10^5^)	52	23 (44)	7.6 (2.8–12.8)	13 (25)	5 (10)	85.0 (53.6–117)
Toulouse	SB (10^5^), CVM (10^5^)	91	28 (31)	9.2 (5.3–17.7)	35 (38)	17 (19)	75.5 (33.0–117)
***Total (n = 413)***		413	157 (38)	8.5 (4.0–16.0)	109 (26)	47 (11)	53.8 (20–98)

*Classified according to the urine collection technique in non-toilet-trained children.

† In colony-forming units/mL.

Abbreviations: Alex for Alexandroupolis; CRP, C-reactive protein; CVM, Clean-voided midstream; IQR, Interquartile range; SA, Suprapubic aspiration; SB, Sterile bag; UC, Urethral catheterization; VUR, Vesicoureteral reflux.

**Figure 1:**
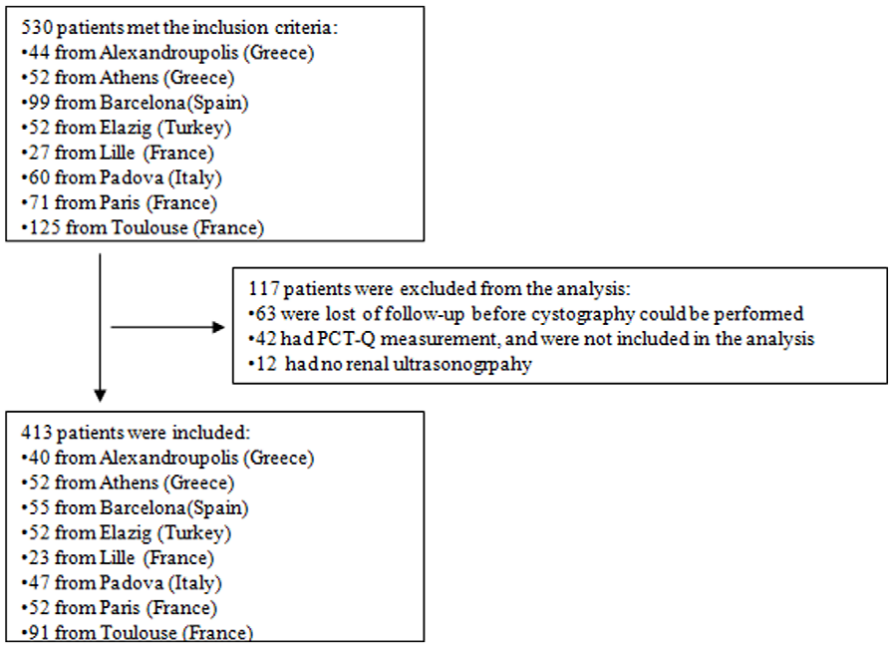
Diagnosis tree and distribution of the study population at each step of the decision rule in the validation population. Abbreviations: PCT, Procalcitonin; VUR, Vesico-ureteral reflux.

Of the 530 who met the inclusion criteria, 417 were finally included in the analysis (Figure 1). The mean age of the children was 11.9 months (SD: 10.7, median: 8.5, Inter-quartile - IQR: 4.0–16.0); 157 (38%) were boys. VUR was diagnosed in 109 (26%) children and 47 (11%) patients had VUR ≥3. No adverse event was reported in performing PCT measurement, renal US nor cystography. Table 1 provides details on the characteristics of each centre population.

### Validity of the rule

**Figure 2:**
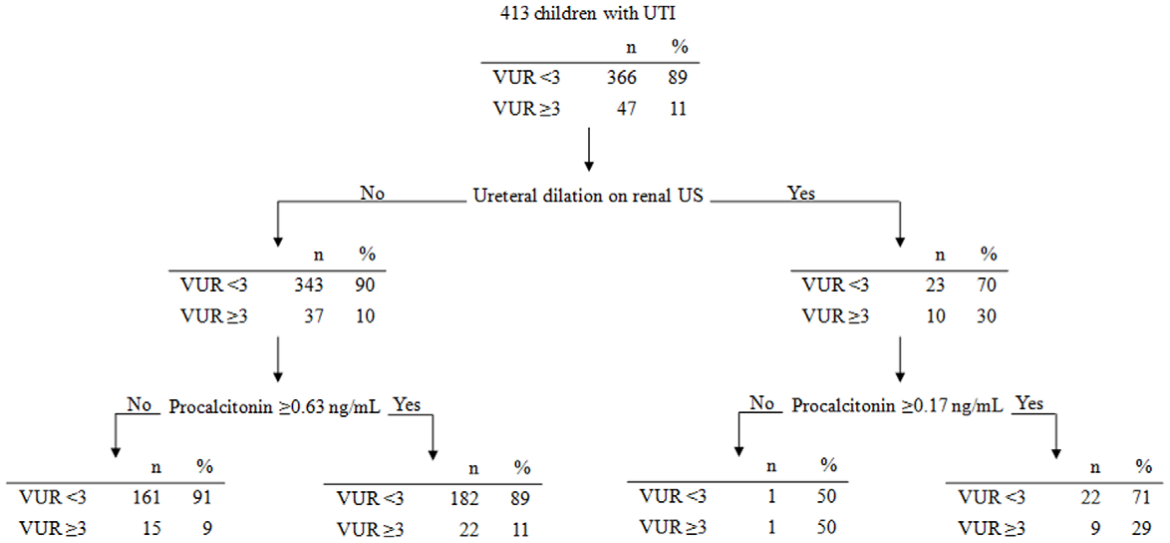
Distribution of Procalcitonin values according to the presence of high-grade VUR and the presence of Ureteral dilation on renal ultrasonography.

**Figure 3:**
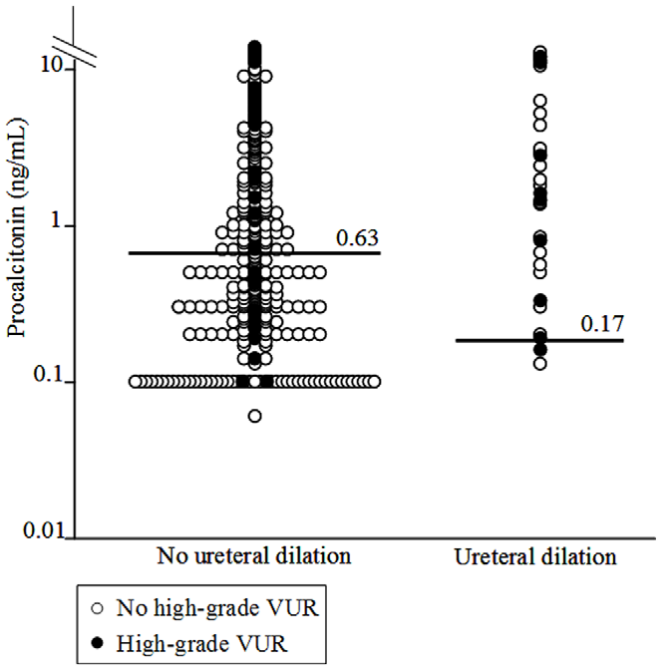
Distribution of Procalcitonin values according to the presence of high-grade VUR and the presence of Ureteral dilation on renal ultrasonography. The horizontal lines are the dichotomization threshold in each group.

When applying the rule to the validation population, 16 (34%) of the 47 patients with VUR ≥3 were misdiagnosed because they presented with either ureteral dilation and PCT <0.17 ng/mL (for one patient – 2%) or without ureteral dilation and PCT <0.63 ng/mL (for the 15–32% - other patients - Figures 2 and 3). We did not find a significant relationship between VUR ≥3 and the clinical decision rule: adjusted OR = 1.5 (95% CI, 0.7–3.4); P = 0.3. On the validation population, the decision rule yielded a 64% (95% CI, 40–76) sensitivity, and a 46% (95% CI, 41–52) specificity (Table 2). Specificity and positive predictive value (46% and 13% respectively) were not significantly different from the derivation set (47%, and 17% respectively), whereas sensitivity and negative predictive value were (64% and 91 respectively for the validation set vs. 86% and 96% respectively for the derivation set-Table 2).

**Table 2:** Sensitivity and specificity of the decision rule in the derivation and validation populations.

	Derivation* (n = 494, prevalence of VUR ≥3: 11%)	Validation (n = 413, prevalence of VUR ≥3: 11%)	Difference†
*Clinical decision rule*			
aOR**	5.2 (2.4–11.3)	1.5 (0.7–3.4)	
Sensitivity	86 (74–93)	64 (50–76)	22 (5 to 38)
Specificity	47 (42–51)	46 (41–52)	0 (−7 to 7)
PPV	17 (13–22)	13 (10–18)	4 (−3 to 10)
NPV	96 (93–98)	91 (86–94)	5 (1 to 11)
*Rounded decision rule*			
aOR**	6.8 (0.9–50.0)	1.3 (0.6–3.2)	
Sensitivity	86 (76–94)	62 (47–74)	26 (9 to 41)
Specificity	44 (40–49)	45 (40–50)	1 (−6 to 8)
PPV	17 (13–21)	13 (9–18)	4 (−2 to 10)
NPV	97 (93–98)	90 (85–94)	6 (1 to 12)
*Rule based on PCT alone*			
aOR**	4.9 (2.3–10.6)	1.3 (0.5–3.4)	
Sensitivity	86 (74–93)	60 (45–72)	28 (11 to 44)
Specificity	45 (40–50)	46 (41–51)	1 (−6 to 8)
PPV	17 (13–21)	12 (9–17)	4 (−2 to 10)
NPV	96 (93–98)	90 (85–93)	7 (2 to 12)

Values are expressed as values or % (95% CI).

Discriminative values were compared using a χ^2^ test for unpaired sample.

*Data in the column come from the previously published derivation of the decision rule [34].

**Adjusted OR were calculated with the multi-level logistic regression models.

† Differences are rounded to the closer integer.

Abbreviations: NPV, Negative predictive value; OR, Odd ratio; PCT, Procalcitonin; PPV, Positive predictive value.

When applying the rounded rule and the rule based on PCT alone to the validation population, we also found a non-significant relationship between VUR ≥3 and the rule, as well as a significant decrease of sensitivity and negative predictive value (Table 2).

In the subgroup of children for whom urine specimens were collected using suprapubic aspiration or urethral catheterization for non-toilet trained children, the relationships between VUR ≥3 and the rules were not significant (Table 3). For the three rules, sensitivities did not differ from those found in the entire population (60 (95% CI, 39–78) in the subgroup *vs.* 64 (95% CI, 50–76) for the entire set for the exact rule), whereas specificities were significantly higher: 58 (95% CI, 51–65) in the subgroup *vs.* 46 (95% CI, 41–52) for the entire set for the exact rule (Table 3).

**Table 3:** Sensitivity and specificity of the decision rule in the whole validation population and in the subgroup of children for whom urines were collected using suprapubic aspiration, urethral catheterization or clean-voided midstream sample.

	Whole population	Subgroup*	Difference
*Clinical decision rule*			
aOR**	1.5 (0.7–3.4)	2.0 (0.4–11.6)	
Sensitivity	64 (50–76)	60 (39–78)	4 (−20 to 28)
Specificity	46 (41–52)	58 (51–65)	11 (2 to 20)
PPV	13 (10–18)	14 (8–22)	1 (−1 to 7)
NPV	91 (86–94)	93 (86–96)	2 (−1 to 8)
*Rounded decision rule*			
aOR**	1.3 (0.6–3.2)	1.9 (0.3–11.1)	
Sensitivity	62 (47–74)	60 (39–78)	2 (−20 to 26)
Specificity	45 (40–50)	55 (48–62)	10 (1 to 19)
PPV	13 (9–18)	13 (8–21)	0 (−1 to 7)
NPV	90 (85–94)	93 (86–96)	2 (−5 to 9)
*Rule based on PCT alone*			
aOR**	1.3 (0.5–3.4)	1.9 (0.3–11.1)	
Sensitivity	60 (45–72)	60 (39–78)	0 (−24 to 25)
Specificity	46 (41–51)	56 (49–63)	10 (1 to 18)
PPV	12 (9–17)	13 (8–22)	−1 (−10 to 7)
NPV	90 (85–93)	93 (86–96)	3 (−5 to 9)

Values are expressed as values or % (95% CI).

Discriminative values were compared using a χ^2^ test for unpaired sample.

*Subgroup of children for who urines were collected using suprapubic aspiration or urethral catheterization.

**Adjusted OR were calculated with the multi-level logistic regression models.

Abbreviations: NPV, Negative predictive value; OR, Odd ratio; PCT, Procalcitonin; PPV, Positive predictive value.

### Comparison of the derivation and validation populations

Because we did not find the similar results between derivation and validation populations, we compared their characteristics. There was no significant difference regarding gender, prevalence of all-grade or high-grade VUR, distribution of age (Table 4). However, CRP was significantly lower in the validation population compared with the derivation population: median = 53.8 mg/L (IQR: 20.0–98.0) *vs.* 77.5 mg/L (IQR: 38.0–140.0), P <0.001. PCT had a trend close to significance to be lower for children with VUR ≥3 in the validation set compared with those in the derivation set: median = 1.5 ng/mL (IQR: 0.3–6.1) *vs.* 2.9 (IQR: 1.2–6.8); P = 0.06. There was also a trend to find more ureteral dilations on renal US in children with VUR <3 in the validation set compared with the derivation one: 23 (6%) *vs.* 16 (4%), P = 0.08.

**Table 4:** Comparison of the characteristics of the derivation and validation populations.

Variables	Derivation* (n = 494, prevalence of VUR ≥3: 11%)	Validation (n = 413, prevalence of VUR ≥3: 11%)	P-value**
Use of sterile bags	238 (48)	214 (52)	0.3
Male gender	197 (40)	157 (38)	0.6
All grade VUR	126 (26)	109 (29)	0.8
High-grade VUR	56 (11)	47 (11)	1.0
Age (months)	12.1 (±11.2); 8.0 (4.0–17.0)	11.9 (±10.7); 8.5 (4.0–16.0)	0.7
CRP (mg/mL)	94.6 (±74.0); 77.5 (38.0–140.0)	68.4 (±64.1); 53.8 (20.0–98.0)	<0.0001
PCT (ng/mL)	4.2 (±19.3); 0.9 (0.3–2.8)	3.5 (±8.6); 0.8 (0.3–3.1)	0.5
in children with VUR <3	3.6 (±19.3); 0.7 (0.3–2.4)	3.4 (±8.8); 0.8 (0.3–2.5)	1.0
in children with VUR ≥3	8.3 (±18.5); 2.9 (1.2–6.8)	4.4 (±6.5); 1.5 (0.3–6.1)	0.06
Ureteral dilation	25 (5)	33 (8)	0.8
in children with VUR <3	16 (4)	23 (6)	0.08
in children with VUR ≥3	10 (18)	20 (21)	0.7

Values are expressed as n (%) for binary variables (gender, All grade and high-grade VUR, Ureteral dilation), and as: mean (±Standard deviation); median (inter-quartile range) for continuous variables (age, CPR, PCT).

*Data in the column come from the previously published derivation of the decision rule [34].

**Binary variables were compared using a χ^2^ test, and continuous variables were compared using the non-parametric Mann-Whitney test.

Abbreviations: CRP, C-reactive protein; PCT, Procalcitonin; U dilation, Ureteral dilation; VUR, Vesico-ureteral reflux.

## Discussion

We report the first attempt to evaluate the reproducibility of the decision rule based on PCT and ureteral dilation proposed by our group [14]. In the derivation study, a significant relationship was found between VUR ≥3 and the rule (P <0.0001), even when considering the rounded rule, or the rule based on PCT only. These significant relationships were not found again in the validation set (P >0.1). The 47% specificity (95%CI, 42–51) of the rule for the prediction of VUR ≥3 was confirmed (46%; 95%CI, 41–52), but not the sensitivity: 60% (95% CI, 50–76) in the validation set vs. 86% (95% CI, 74–93) in the derivation population. The results were similarly comparable between validation and derivation populations for the rounded rule and the rule based on PCT only. Applying the rule to the validation set, we would not have prescribed cystography, and then misdiagnosed VUR in 16 patients (15 without ureteral dilation), representing 34% of the patients with VUR ≥3. The rule only had missed 9 (16%) children among those with VUR ≥3 in the derivation study [14].

The first issue to be addressed to investigate the decreases of predictive ability of a rule is the difference in the derivation and validation populations, which would explain why a decision rule could not be transferred across those sets of patients [21]. In the present case, populations were not significantly different (P >0.05) for the classic parameters: gender, prevalence of all-grade and high-grade of VUR. Nevertheless, the validation population had a significantly lower level of inflammatory biomarkers: for CRP for all children, in PCT was also lower in children with VUR ≥3. This result means that the entire distribution of PCT values was moved towards lower values for children with high-grade VUR in the validation population; it could explain why the group of 15 (94%) out of 16 patients with VUR≥3 were missed by the rule, and thus belonged to the rule branch of “patients without ureteral dilation and PCT <0.63 ng/mL”. As the weight of the rule is mainly carried by PCT, a lowest distribution of PCT values might have a major influence on the results of the rule validation set; it is demonstrated by that 15 (94%) out of the 16 patients missed by the rule were not rescued by ureteral dilation criterion. Indeed, this hypothesis also may explain why even the rule based on PCT alone failed to reproduce the 85% sensitivity, even though this result had previously been validated in two multicentre cohort studies [16], [17]. This significant difference in inflammatory biomarkers distributions between validation and derivation sets could be due to the fact that samples were collected at different time points during UTI course. We were not able to verify this suggestion, because the centres identified the exact time of inflammatory markers measurement from the appearance of fever, and this data was not collected. Further study of this time interval would be necessary to improve this rule and to implement it safely. These differences regarding the inflammatory parameters distributions were unpredictable before the study validation, because all centres were European and applied the same standard procedures to diagnose and treat children with UTI. Moreover, there was also a trend (p = 0.08) in the difference between derivation and validation sets on the ureteral dilation on renal US number in children with VUR <3. This finding may also have added to the differences between the two populations concerning the key variables of the rule. Furthermore, we acknowledge that the rule included ureteral dilation, which is a renal US criterion with no measurement of its inter-operator variability in a multi-case multi-reader study, even if it was found to be the best US renal criterion to predict high-grade VUR [18]. This weakness of the rule needs to be evaluated and corrected.

The second issue concerning the validation's difficulties to reproduce derivation results is the limitations of the external validation study. The validation was a secondary analysis of previously published prospective cohort studies, as was the derivation study. Because our group performed a systematic review and meta-analysis on PCT in UTI in children, we gathered the worldwide published data of children with PCT and VUR [20]. The derivation study was based on the initially published cohort studies, while the validation study was premised on the later ones. We did not, however, believe that the structure of these studies would affect the quality of the data or introduce a bias.

The use of sterile bags for urine collection for the non-toilet trained children in half of the centres might have introduced a selection bias, because this technique is less specific than suprapubic aspiration or urethral catheterization [1]. However, there was no significant difference in the number of children for whom urine were collected by sterile bags, compared with those in the derivation population: 238 (48%) children vs. 214 (52%), P = 0.3 (Table 4). Interestingly, the specificities of the rules were significantly higher in the subgroup of children for whom urine specimens were collected properly than in the whole population. This result can be explained by the lower specificity for UTI diagnosis of sterile bags compared with recommended techniques. Indeed, because VUR is known to be a risk factor for UTI, the use of sterile bags might have increased the number of children with VUR <3 more than those with VUR ≥3. In the same manner, because PCT is positively correlated with the presence and severity of UTI [20], and because the weight of the rule was more carried by PCT than by ureteral dilation [14], the use of sterile bags may have increased the number of children with a negative result than the one with a positive one. Nevertheless, some centres (e.g. Padova, Italy), in order to decrease the likelihood of false positive results due to bag urine collection, included only children with two consecutive positive urine cultures. The combined increases (in the number of children with VUR <3 and in the number of children with PCT <0.5 ng/mL when sterile bags are used to collected urine) resulted in an underestimate in the specificity in children for whom urine were collected by sterile bags, and thus accounts for the significant difference in specificities. It did not, however, explain the failure to reproduce the sensitivity of the rule, nor the loss of a significant relationship between VUR ≥3 and the rules. To summarize, the validation study may have had some limitations, but none of these limitations appeared to explain fully the failure to validate externally the decision rule.

The validation of the decision rule based on PCT and ureteral dilation on renal US confirmed the rule specificity, but showed a loss of its sensitivity, which led to a misdiagnosis of 34% of children with VUR ≥3. The fact that the rule performed better in the derivation population than in the validation one was predictable, according to the Evidence Based Working Group [21]. However, the decrease in sensitivity might also be primarily due to differences regarding PCT distributions between derivation and validation sets and was thus unpredictable prior to the study. The rule therefore may need greater refinement, particularly regarding the time of PCT measurement and the variability between observers of ureteral dilation. Furthermore, the outcome should also be reconsidered and modified for a composite outcome, including high-grade VUR and renal scars, which are precisely the cause of kidney injuries leading to future complications, and the real goal of any nephroprotection strategy including VUR treatment.

## Methods

### Study design

We conducted a secondary analysis of already published data on children with UTI, PCT and VUR. Because we had conducted a systematic review and meta-analysis on individual patient data on early and late renal scarring and PCT in children with UTI [16], we knew all the centres possessing data on PCT and VUR in children with UTI. To perform the external validation of the rule, we included all centres [18], [22]–[31] that had not been included in the study to derive the rule [14]. All consecutive children in each center, aged one month to four years and admitted with a first febrile UTI (temperature ≥38°C with a positive bacterial urine culture defined according to each centre criteria – Table 1) were considered for inclusion. Children with an already known uropathy (ie. any urological or urinary tract abnormality, as posterior urethral valves, ectopic ureter, VUR); at the time of the UTI diagnosis, those who had received antibiotics in the 48 hours before diagnosis, and those who had undergone radionuclide cystography (which does not distinguish high-grade from low-grade VUR [32]) were not included. Of note, results of antenatal renal US were not taken into account to include children, insofar as there was no post-natal diagnostic confirmation before UTI diagnosis. Because this study was a secondary analysis of already-published prospective cohort studies, the electronic data files were merged across studies; informed consent had had already been obtained for the initial studies.

### Outcome definition

All patients had a voiding cystography, the gold standard examination for the diagnosis and classification of VUR. Cystographies were read according to the “International System of Radiological Grading of Vesicoureteric Reflux” [33] by an experienced senior radiologists in Pediatrics in each centre, and blinded to PCT and renal US results. Moderate and high-grade VUR was a priori defined as a VUR grade ≥3, which means that VUR extends up to the renal pelvis with an increased ureteral dilation with the grade of reflux [7].

### Definition of predictors included in the rule

Each child's serum PCT was prospectively measured at admission for febrile UTI (i.e. when children arrived at the Emergency Department), with the LUMItest PCT immunoluminometric assay (BRAHMS, Hennigsdorf, Germany). The other potential predictive variables came from the findings of renal US performed at the time of UTI diagnosis by an experienced senior pediatric radiologist blinded to the PCT measurement, and before cystography was performed: ureteral dilation (defined by ureter visibility during renal US). The imaging studies were reviewed blinded to cystography results, and data were extracted from the radiologist's record.

### Statistical analyses

We first described the study population's general characteristics. We then applied the rule to every patient, classifying each one as cystography recommended or not. We also applied the rounded rule (i.e. a cystography should performed in case of ureteral dilation and PCT ≥0.2 ng/mL, or if PCT ≥0.6 ng/mL in absence of ureteral dilation), and the rule based on PCT alone (a cystography should be prescribed if PCT ≥0.6 ng/mL) to each child. We evaluated the relationship between VUR ≥3 and the decision rules with an adjusted OR using a multi-level logistic regression model (where centers were considered as the group level variable). We then calculated for the clinical decision rule, the rounded rule, and the rule based on PCT alone, the sensitivity, specificity, positive and negative predictive values. We ran the same calculation in the subgroup of children for whom urine specimens were collected using “recommended” techniques (i.e. suprapubic aspiration or urethral catheterization for non-toilet trained children and clean-voided midstream for toilet trained children). Discriminative values were compared with those of the derivation population [14]. In case of significant difference, we compared the characteristics of derivation and validation populations using χ^2^ tests and non-parametric Mann-Whitney tests. Statistical analyses were performed with Stata 11/SE software (StataCorp, College Station, TX, USA) and Confidence Interval Analysis software (London, UK).
