# Correction: Prediction of High-Grade Vesicoureteral Reflux after Pediatric Urinary Tract Infection: External Validation Study of Procalcitonin-Based Decision Rule

**DOI:** 10.1371/annotation/c23484a1-f516-4391-a23b-3db456be8edf

**Published:** 2012-02-07

**Authors:** Sandrine Leroy, François Bouissou, Anna Fernandez-Lopez, Metin K. Gurgoze, Kyriaki Karavanaki, Tim Ulinski, Silvia Bressan, Geogios Vaos, Pierre Leblond, Yvon Coulais, Carlos Luaces Cubells, A. Denizmen Aygun, Constantinos J. Stefanidis, Albert Bensman, Liviana Da Dalt, Stefanos Gardikis, Sandra Bigot, Dominique Gendrel, Gérard Bréart, Martin Chalumeau

There was a spelling error in the fifteenth author's name. The correct spelling is Liviana Da Dalt.

